# Endokavitäre Kontakttherapie vs. EBRT als Boost bei organerhaltender Strahlentherapie von T2–3-Rektumkarzinomen: Ergebnisse der OPERA-Studie

**DOI:** 10.1007/s00066-023-02112-7

**Published:** 2023-06-26

**Authors:** Emilie von Wieding, Jürgen Dunst

**Affiliations:** Klinik für Strahlentherapie/Radioonkologie, UKSH, Campus Kiel, Kiel, Deutschland

## Hintergrund

Organerhaltende Therapiekonzepte sind bei tiefsitzenden Rektumkarzinomen durchaus möglich und werden aktuell in zahlreichen Studien weiter optimiert [3, 4]. Dabei wird auf eine geplante Operation (die meistens eine Exstirpation mit dauerhaftem Anus praeter bedeuten würde) verzichtet, wenn nach neoadjuvanter Radiochemotherapie eine komplette Remission erreicht wird. Es gibt Hinweise für eine Dosis-Wirkungs-Beziehung der Radiotherapie in diesen Konzepten [1]. Die Dosiseskalation der Radiotherapie ist deshalb grundsätzlich sinnvoll mit dem Ziel, die Radiochemotherapie als definitive Therapie zu etablieren. Die französische OPERA-Studie liefert wichtige Hinweise für die weitere Entwicklung dieser Konzepte [5].

## Methodik

OPERA war eine multizentrische, offene, randomisierte, kontrollierte Phase-3-Studie, die an 17 Einrichtungen in Frankreich, Großbritannien und der Schweiz durchgeführt wurde. Acht Einrichtungen (4 × UK, 3 × Frankreich, 1 × in der Schweiz) verfügten über die Möglichkeit der endokavitären Kontakttherapie (nebenbei: Wie die Randomisierung und Behandlung vom Patienten an den anderen neun Einrichtungen ohne Kontakttherapie erfolgte, ist nicht genau beschrieben). Eingeschlossen wurden operable erwachsene Patienten mit cT2–3b-Adenokarzinomen des unteren und mittleren Rektums. Der maximale Tumordurchmesser war < 5 cm, und in der Bildgebung musste eine cN0-Situation vorliegen bzw. durften suspekte LK maximal 8 mm groß sein. Alle Patienten erhielten eine neoadjuvante Radiochemotherapie, also externe Strahlentherapie (EBRT) mit 45 Gy in 25 Fraktionen, simultan Capecitabin in typischer Dosierung (2 × täglich 825 mg/m^2^). Alle Patienten erhielten einen Boost, der nach 1:1-Randomisierung entweder eine externe Strahlentherapie mit 5 × 1,8 Gy (Gruppe A) war oder ein Boost mit intrakavitärer Kontaktbrachytherapie (3 × 30 Gy Oberflächendosis mit dem Papillon-Applikator, Gruppe B). Der Boost erfolgte in Gruppe A nach der EBRT, in Gruppe B bei Tumoren < 3 cm vor der EBRT und sonst ebenfalls nach der EBRT. Stratifizierungen erfolgten nach Studienzentrum, T‑Kategorie (cT2 vs. cT3a oder cT3b) und Tumorlage (< 6 cm vom Analrand vs. ≥ 6 cm). Primärer Endpunkt war der Organerhalt nach 3 Jahren.

## Ergebnisse

Zwischen Juni 2015 und Juni 2020 wurden 148 Patienten rekrutiert. 141 Patienten wurden in die primäre Wirksamkeitsanalyse einbezogen, darunter 69 der Gruppe A (29 mit Tumoren < 3 cm Durchmesser und 40 mit Tumoren ≥ 3 cm) und 72 der Gruppe B (32 mit Tumoren < 3 cm und 40 mit Tumoren ≥ 3 cm). Nach einer medianen Nachbeobachtungszeit von 38 Monaten betrug die 3‑Jahres-Organerhaltungsrate 59 % in Gruppe A gegenüber 81 % in Gruppe B (Hazard Ratio 0,36; *p* = 0,0026). Bei Patienten mit Tumoren von weniger als 3 cm Durchmesser betrug die 3‑Jahres-Organerhaltungsrate 63 % in Gruppe A gegenüber 97 % in Gruppe B (HR 0,07; *p* = 0,012), bei Patienten mit Tumoren größer 3 cm 55 % gegenüber 68 % (HR 0,54; *p* = 0,11). Unerwünschte Ereignisse von Grad 2–3 traten bei 30 % der Patienten in Gruppe A und bei 42 % in Gruppe B auf, vor allem akute Proktitis (6 % in Gruppe A, 13 % in Gruppe B) und akute Hautreaktion (10 % in Gruppe A, 3 % in Gruppe B). Die wichtigste Spätfolge waren rektale Blutungen von Grad 1–2 (12 % in Gruppe A vs. 63 % in Gruppe B, *p* < 0,0001), die überwiegend nach 3 Jahren abklangen.

## Schlussfolgerungen der Autoren

Ein hoch dosierter Boost mittels intrakavitärer Kontakttherapie als Ergänzung zur neoadjuvanten Radiochemotherapie verbesserte signifikant die 3‑Jahres-Organerhaltungsrate, insbesondere bei Patienten mit Tumoren kleiner als 3 cm.

## Kommentar

Das ist eine exzellente Arbeit aus der Arbeitsgruppe von Jean Pierre Gerard, einem DEGRO-Ehrenmitglied, und auch wenn die etwas ungewöhnliche Technik einer schnellen Verbreitung dieses Therapiekonzepts entgegensteht, zeigt die Studie nach unserer Auffassung, in welche Richtung sich die Therapie des Rektumkarzinoms weiterentwickeln kann. Folgende Aspekte sollte man in der Diskussion berücksichtigen:

1. Es gibt Hinweise dafür, dass die Remissionsrate nach neoadjuvanter Radiochemotherapie mit zunehmender Strahlendosis steigt [1]. Die Datenlage in randomisierten Studien ist aber unklar [2]. Diese Studie ist erstmals ein klarer und eindrucksvoller Beweis, dass mit sehr viel höheren Dosen auch sehr viel höhere Remissionsraten erreicht werden können.
2. Die intrakavitäre Kontakttherapie des Rektumkarzinoms wurde in den 1970er-Jahren in Frankreich von Papillon, einem der renommiertesten Radioonkologen seiner Zeit, entwickelt und damals vor allem als alleinige Therapie bei kleinen Rektumkarzinomen zum Organerhalt eingesetzt [8]. Das Therapieprinzip hat sich aber nie auf breiter Basis etabliert. Das hier verwendete Gerät ist ähnlich, und die Applikation erfolgt über ein peranal eingeführtes Rohr. Geräte dieser Art sind nur an wenigen Standorten verfügbar. Ob diese Technik, wie von ihren Protagonisten behauptet, eine Renaissance erleben wird, darf man kritisch hinterfragen [6, 7].
3. Unabhängig von der Technik kann man aber feststellen, dass in dieser Studie exzellente Ergebnisse hinsichtlich lokaler Tumorfreiheit und Organerhalt erreicht wurden. Daraus ein Alleinstellungsmerkmal für die intrakavitäre Boosttechnik abzuleiten, ist sicher nicht gerechtfertigt, denn der Erfolg ist durch die hohe Dosis zu erklären. Die verabreichten drei Fraktionen mit jeweils 30 Gy Oberflächendosis kann man getrost als ablative Strahlentherapie bezeichnen.
4. Die wichtigste daraus resultierende Frage ist daher, ob man mit modernen Techniken einen ähnlichen strahlenbiologischen Effekt erreichen kann. Diese etwas archaisch anmutende Technik hat aus unserer Sicht einige Nachteile. Durch den starren, peranal eingeführten Applikator sind nicht alle Lokalisationen gut zu erreichen. Eine 3‑D-Dosisberechnung ist nicht möglich. Wegen des steilen Dosisabfalls ist eine sehr inhomogene Dosis im Tumor anzunehmen, und das gilt sicher auch für das gesunde Gewebe außerhalb des Tumors. Bei kleinen Tumoren (hier bis 3 cm) kann man grob schätzen, dass in einem kugeligen oder ellipsoid konfigurierten Tumorvolumen mediane Dosen von 10 Gy pro Fraktionen erreicht werden. Dass ein solcher Boost (3 × 10 Gy) gut wirkt, verwundert nicht. Bei flachen Tumoren sind hohe Dosen in der Rektumschleimhaut unvermeidbar; das erklärt die hohe Rate an Teleangiektasien. Sehr positiv fällt aber auf, dass keine Spätkomplikationen in Form von Fisteln oder Perforationen beobachtet wurden. Wenn man alle diese Aspekte bewertet, sollte es aber möglich sein, im Zeitalter der bildgeführten adaptiven Strahlentherapie gleichwertige oder bessere Dosisverteilungen zu erreichen.

## Fazit

Eine radikale Dosiseskalation bringt eindeutige Vorteile bezüglich Tumorkontrolle und Organerhalt. Die Optimierung dieses Konzepts ist eine spannende und wichtige Herausforderung.

*Emilie von Wieding, Jürgen Dunst, Kiel*
